# Association of Thrombosis With Hypereosinophilic Syndrome in Patients With Genetic Alterations

**DOI:** 10.1001/jamanetworkopen.2021.19812

**Published:** 2021-08-06

**Authors:** Orly Leiva, Olesya Baker, Andrew Jenkins, Andrew M. Brunner, Hanny Al-Samkari, Rebecca Karp Leaf, Rachel P. Rosovsky, Amir T. Fathi, James Weitzman, Larissa Bornikova, Valentina Nardi, Gabriela S. Hobbs

**Affiliations:** 1 Department of Medicine, Brigham and Women’s Hospital and Harvard Medical School, Boston, Massachusetts; 2 Center for Clinical Investigation, Brigham and Women’s Hospital, Boston, Massachusetts; 3 Division of Hematology Oncology, Department of Medicine, Massachusetts General Hospital and Harvard Medical School, Boston

## Abstract

**Importance:**

Hypereosinophilic syndromes (HESs) are a rare group of disorders that result in overproduction of eosinophils, leading to tissue damage. Thrombotic complications in HES and associated risk factors in this patient population have not been extensively studied.

**Objective:**

To investigate the rates of and risk factors associated with thrombotic events in patients with HES, including markers of clonal hematopoiesis as evidenced by molecular aberrations on next-generation sequencing.

**Design, Setting, and Participants:**

This retrospective cohort study evaluated patients seen at Brigham and Women’s Hospital and Harvard Medical School in Boston, Massachusetts, from January 1, 2015, to January 1, 2020. Patients who had hypereosinophilia with an absolute eosinophil count of 1500 cells/μL or greater on 2 separate occasions at least 1 month apart and who underwent genetic or molecular testing as part of their work-up were included. Patients with secondary eosinophilia were excluded.

**Main Outcomes and Measures:**

Symptomatic and asymptomatic arterial and venous thrombotic events after the diagnosis of HES and all-cause death.

**Results:**

A total of 71 patients (median age, 58 years [interquartile range (IQR), 43-67 years]; 36 women [51%]; 57 White patients [80%]) were included. Patients had a median follow-up time of 29 months (IQR, 19-49 months). Seventeen patients (24%) had 1 or more thrombotic events, including 11 venous thromboembolic events and 11 arterial thrombotic events (8 patients had ≥1 event and 3 patients had recurrent events). Patients with 1 or more thrombotic events had a higher median Eastern Cooperative Oncology Group performance status (median, 1 [IQR, 1-2] vs 0 [IQR, 0-1]; *P* = .002), had more frequent cardiac involvement (7 of 17 events [41%] vs 6 of 54 events [11%]; *P* = .01), more frequently received treatment (17 of 17 events [100%] vs 40 of 54 events [74%]; *P* = .02), and had more molecular aberrations on next-generation sequencing (12 of 17 [71%] vs 12 of 54 [26%]; *P* = .003) vs patients without thrombosis. After multivariable analysis, the presence of molecular aberration was associated with increased odds of thrombosis (adjusted odds ratio, 5.4; 95% CI, 1.1-27.7). Death occurred more frequently in patients with thrombotic events compared with those without (6 of 17 [35%] vs 2 of 54 [4%]; *P* = .002) and in patients with molecular aberrations compared with those without (6 of 24 [25%] vs 1 of 40 [3%]; *P* = .009), although only thrombotic events were significantly associated with increased odds of death after multivariable analysis.

**Conclusions and Relevance:**

In this cohort study, thrombosis was common in patients with HES and was significantly associated with increased risk of death.

## Key Points

**Question:**

What are the incidence of and risk factors associated with thrombotic events in patients with hypereosinophilic syndromes?

**Findings:**

In this cohort study that included 71 adults with hypereosinophilic syndrome, thrombotic events were common with 24% experiencing at least 1 event, which was associated with increased risk of death. The presence of molecular aberration on next-generation sequencing was associated with a 5-fold increase in risk of thrombosis.

**Meaning:**

In this study, hypereosinophilic syndromes, particularly those with molecular aberrations, were associated with high rates of thrombosis; further investigation on the utility of thromboprophylaxis in this patient population is warranted.

## Teaser

This cohort study investigates the incidence of and risk factors associated with thrombotic events in patients with hypereosinophilic syndromes.

## Introduction

Hypereosinophilic syndromes (HESs) with or without identifiable clonal markers are a heterogeneous group of hematologic disorders characterized by the overproduction of eosinophils, leading to tissue eosinophilic infiltration and damage.^1^ Case reports have described thrombotic complications in HES, and 1 retrospective cohort study of 138 patients with HES reported thrombotic events in 21% of patients.^2,3,4,5,6^ These observations are supported by preclinical studies implicating increased tissue factor expression in peripheral eosinophils in patients with HES.^7^ Additionally, eosinophil granules contain inflammatory, oxidative, and prothrombotic components, such as major basic protein and eosinophil peroxide, that may also increase the risk of thrombosis.^7,8,9,10^ However, to our knowledge, the risk factors for thrombosis in patients with HES have not been investigated.

Hypereosinophilic syndromes may be driven by rearrangements or variations in different genes (*PDGFRA* [OMIM 173490], *PDGFRB* [OMIM 173410], *FGFR1* [OMIM 608858], *PCM1* [OMIM 600299], or *JAK2* [OMIM 147796]) or harbor individual variations in genes associated with clonal hematopoiesis (*TET2* [OMIM 612839], *RUNX1* [OMIM 151385], *EZH2* [OMIM 601573], *DNMT3A* [OMIM 602769], *NOTCH1* [OMIM 190198], and *SETBP1* [OMIM 611060]), although often no genetic aberration is found.^1,11,12,13^ Several hematologic disorders, including clonal hematopoiesis of indeterminate potential (CHIP) and myeloproliferative neoplasms, are associated with increased thrombotic risk, but the incidence of these events in HES is understudied.^14^ The presence of CHIP with variations in *DNMT3A*, *TET2*, *ASXL1* (OMIM 612990), and *JAK2* in healthy patients is associated with increased risk of death and cardiovascular events (including arterial thrombotic events such as myocardial infarction).^15^ Given that these somatic variations have been identified in patients with HES, we hypothesized that the presence of genetic abnormalities is associated with an increased risk of thrombotic events in patients with HES.

## Methods

### Patients

We used the Research Patient Data Registry, a registry that extracts data from several hospitals in Massachusetts General Brigham health system, including Massachusetts General Hospital, to identify patients to include in this cohort study. We selected patients who received care at Massachusetts General Hospital between January 1, 2015, and January 1, 2020, and had absolute eosinophil count of 1500 cells/μL (to convert to ×10^9^ per liter, multiply by 0.001) or greater on 2 separate occasions at least 1 month apart (n = 328).^16^ Patients with HES who had molecular evaluation with either RNA- or DNA-based next-generation sequencing (NGS) assays that analyze for common gene variations seen in hematologic malignant neoplasms (Rapid Heme Panel [Dana Farber Cancer Institute]; Heme SnapShot [Massachusetts General Hospital]), an RNA-based NGS assay (Heme fusion [Massachusetts General Hospital]), cytogenetics, or fluorescent in situ hybridization assay as part of their work-up for HES were included in our cohort. Patients who were younger than 18 years (n = 8); had eosinophilia due to another hematologic malignant or myeloproliferative neoplasms, or who had undergone bone marrow transplant unrelated to HES (N = 211); had secondary eosinophilia including eosinophilic granulomatosis with polyangiitis (n = 33); or did not have molecular testing (n = 5) were excluded (total excluded, n = 257) (eFigure 1 in the Supplement).^17^ The study protocol was approved by the Massachusetts General Brigham institutional review board, which granted a waiver of informed consent owing to the retrospective nature of the study wherein no procedure or intervention was performed on the patients. A complete medical history was obtained from the electronic medical record and including patient demographic details, cardiovascular risk factors, medical history, laboratory values, relevant imaging, and medications prescribed at the time of HES diagnosis. Spleen size (largest anterior-posterior measurement on axial computed tomographic [CT] imaging) at the time of HES diagnosis (within 6 months) was obtained from patients who had undergone CT imaging. Our study followed the Strengthening the Reporting of Observational Studies in Epidemiology (STROBE) reporting guideline.

### Organ Involvement Definitions

Organ involvement in HES was defined as having at least 1 organ system affected by the disease (pulmonary, gastrointestinal, skin, cardiac, hepatic, or other). Patients were deemed to have pulmonary involvement if there were parenchymal lung changes on cross-sectional imaging (eg, ground glass opacities) or eosinophils on bronchoalveolar lavage. Gastrointestinal and skin involvement were defined as biopsy-proven eosinophilic infiltration of the gastrointestinal tract or skin, respectively. Patients were deemed to have hepatic involvement if they had elevations in their hepatic enzyme (alanine aminotransferase, aspartate aminotransferase, alkaline phosphatase) at the time of HES diagnosis. Cardiac involvement was defined as at least 1 of the following: troponin elevation at the time of HES diagnosis, cardiac magnetic resonance imaging findings consistent with myocarditis, clinical symptoms of myocarditis, new unexplained heart failure at the time of HES diagnosis, or biopsy-proven eosinophilic infiltration.

### Thrombotic Event Definitions

Symptomatic and asymptomatic thrombotic events were included. All venous thromboembolic events (VTEs) and arterial thrombotic events (ATEs) were confirmed using diagnostic imaging techniques, including ultrasound, CT, or magnetic resonance imaging. Venous thromboembolic events were deemed to be provoked if a major transient risk factor occurred within 3 months before a VTE (eg, hospitalization, surgery, and oral combined contraceptive use).^18^ Cross-sectional brain imaging was used to confirm ischemic stroke; diagnosis of transient ischemic attack made by a neurologist was used to confirm transient ischemic attack events. Diagnosis of myocardial infarction or acute coronary syndromes (including unstable angina) was made by clinical criteria, cardiologist diagnosis, elevation in biomarkers, and electrocardiographic changes. The thrombotic events outcome was a composite of all VTEs and ATEs.

### Statistical Analysis

Categorical variables were compared using the Fisher exact test, and continuous variables were compared using a 2-sample *t* test. Multivariable analysis of factors associated with thrombosis was performed by logistic regression using age, cardiovascular disease (CVD) (including atrial fibrillation, heart failure, and atherosclerosis), Eastern Cooperative Oncology Group performance (ECOG) status, hypertension, aspirin use, cardiac HES involvement, and molecular aberration status as variables. Multivariable analysis of the association of thrombosis and presence of molecular aberration with death was performed by logistic regression using age, CVD, ECOG status, age, thrombosis, and molecular aberration as covariables. Kaplan-Meier curves for thrombosis-free and death-free survival were compared between molecular aberration and no aberration groups using log-rank test. Statistical analysis was performed using Stata, version 15.1 (StataCorp LLC), GraphPad Prism 8 (GraphPad Inc), and Microsoft Excel 360 (Microsoft Corp). All *P* values were 2-sided, and *P* < .05 was considered statistically significant.

## Results

### Patient Characteristics and Thrombotic Risk Factors

A total of 71 patients (median age, 58 years [interquartile range (IQR), 43-67 years]; 36 women [51%]; 57 White [80%]) were included in this cohort study. Patients had a median follow-up time of 29 months (IQR, 19-49 months). Seventeen patients (24%) with HES had at least 1 thrombotic event, including 11 VTEs (15%) and 11 ATEs (8 patients had ≥1 event; 3 patients had recurrent events) (Table 1). There was no difference in body mass index, smoking status, Charlson Comorbidity Index, previous VTE, diabetes, and chronic kidney disease between patients with and without thrombotic events (Table 1). A history of CVD (including heart failure, atrial fibrillation, and atherosclerosis) (9 of 17 patients [53%]) and hypertension (10 of 17 patients [59%]) was more prevalent in patients with thrombotic events compared with those without such events (CVD history, 15 of 54 patients [28%]; hypertension history, 17 of 54 patients [31%]), although this difference was not statistically significant. Patients with 1 or more thrombotic events had a higher median ECOG performance status (median [IQR], 1 [1-2] vs 0 [0-1]; *P* = .002), had more frequent cardiac involvement (7 of 17 [41%] vs 6 of 54 [11%]; *P* = .01), more frequently received treatment (17 of 17 [100%] vs 40 of 54 [74%]; *P* = .02), and had more molecular aberrations on NGS (12 of 17 [71%] vs 12 of 54 [26%]; *P* = .003) vs patients without thrombosis (Table 2), although left ventricular ejection fraction (LVEF) was not different between the 2 groups (with thrombotic events, 62% LVEF [12% patients] vs no thrombotic events, 61% LVEF [11% patients]). Aspirin use at the time of HES diagnosis was also higher among patients with a thrombotic event (9 of 17 patients [53%]) compared with those without such an event (12 of 54 patients [22%]; *P* = .03). Significantly more patients with thrombotic events died (6 of 17 patients [35%]) compared with patients without such events (2 of 54 patients [4%]; *P* = .002).

**Table 1.** Patient Characteristics and Thrombotic Events

Characteristic	No. (%)			*P* value
	All (N = 71)	Thrombotic event		
		Yes (n = 17)	No (n = 54)	
Sex				
Female	36 (51)	6 (35)	30 (56)	.46
Male	35 (49)	9 (65)	24 (44)	
Age at HES diagnosis, median (IQR), y	58 (43-67)	60 (43-65)	55.5 (42-68)	.81
Follow-up, median (IQR), mo	29 (19-49)	35 (18-52)	27 (20-49)	.69
BMI, mean (SD)	27.3 (6.7)	29.7 (6.7)	26.6 (6.6)	.09
ECOG performance status, median (IQR)	0 (0-1)	1 (1-2)	0 (0-1)	.002
Smoking status				
Never	41 (58)	10 (59)	31 (57)	.71
Former	26 (37)	6 (35)	20 (37)	.70
Current	4 (6)	1 (6)	3 (6)	>.99
CCI, median (IQR)	2 (0-4)	3 (2-5.5)	2 (0-3)	.16
Comorbidities				
CVD^a^	24 (34)	9 (53)	15 (28)	.06
Diabetes	17 (24)	5 (29)	12 (22)	.54
Hypertension	27 (38)	10 (59)	17 (31)	.05
Previous VTE	7 (10)	2 (12)	5 (9)	.76
CKD	16 (23)	5 (29)	11 (20)	.44
Anticoagulation use	10 (14)	2 (12)	8 (15)	.75
Aspirin use	21 (30)	9 (53)	12 (22)	.03
Outcome				
Death	8 (11)	6 (35)	2 (4)	.002
Thrombotic events				
Any	17 (24)	17 (100)	NA	NA
VTE	11 (15)	11 (65)		
ATE	8 (11)	8 (47)		
Recurrent (% of patients with events)	3 (18)	3 (18)		
Time to first event, median (IQR), mo	10 (5-27)	10 (5-27)		
VTE (% of events)				
DVT	6 (55)	6 (55)	NA	NA
PE	5 (45)	5 (45)		
Recurrent	0	0		
Provoked^b^	5 (45)	5 (45)		
Arterial events (% of events)				
MI	1 (12)	1 (12)	NA	NA
Stroke or TIA	4 (50)	4 (50)		
Peripheral ATE	2 (25)	2 (25)		
Intracardiac thrombus	1 (12)	1 (12)		

Abbreviations: ATE, arterial thrombotic event; BMI, body mass index (calculated as weight in kilograms divided by height in meters squared); CCI, Charlson Comorbidity Index; CKD, chronic kidney disease; CVD, cardiovascular disease; DVT, deep venous thrombosis; ECOG, Eastern Cooperative Oncology Group; HES, hypereosinophilic syndrome; IQR, interquartile range; MI, myocardial infarction; NA, not available; PE, pulmonary embolism; TIA, transient ischemic attack; VTE, venous thromboembolic event.

^a^ Cardiovascular disease includes atrial fibrillation, heart failure, and atherosclerotic disease.

^b^ Provoked venous thromboembolism occurred in the setting of hospitalization, oral combined contraceptive use, and indwelling central venous catheter.

**Table 2.** Hypereosinophilic Syndrome Disease Characteristics and Thrombosis

Characteristic	No. (%)			*P* value
	All (N = 71)	Thrombotic event		
		Yes (n = 17)	No (n = 54)	
Organ involvement	60 (85)	16 (84)	44 (81)	.21
Cardiac	13 (18)	7 (41)	6 (11)	.01
Treatment				
Any	57 (80)	17 (100)	40 (74)	.02
Steroids	51 (72)	15 (88)	36 (67)	.12
Imatinib	7 (10)	1 (6)	6 (11)	.99
Hydroxyurea	10 (14)	5 (29)	5 (9)	.05
Anti–interleukin-5	19 (27)	7 (41)	12 (22)	.21
Other	13 (18)	4 (24)	8 (15)	.44
NGS testing performed	64 (90)	17 (100)	47 (87)	.18
Molecular aberrations on NGS (% of tested)				
Any	24 (34)	12 (71)	12 (26)	.003
*PDGFRA/B* or *FGFR1*^a^	4 (6)	0	4 (9)	.57
CHIP-associated gene^b^	8 (13)	5 (29)	3 (6)	.03
* JAK2*	2 (3)	2 (12)	0	.07
* TET2*	2 (3)	2 (12)	1 (2)	.17
* RUNX1*	3 (5)	2 (12)	0	.07
* DNMT3A*	3 (5)	1 (6)	2 (4)	.99
Splicing genes^c^	5 (8)	5 (29)	0 (0)	<.001
Other	15 (23)	9 (53)	6 (13)	.14
>1 Variation (% of those with variation)	7 (29)	7 (58)	0	.002
VAF, mean (SD), %^d^	36.8 (23.3)	44.2 (25.1)	26.6 (17.1)	.11
LT or BMT for HES	2 (3)	1 (6)	1 (2)	.42
Studies at diagnosis of HES, mean (SD)				
AEC, cells/μL^e^	4.1 (4.5)	2.7 (1.5)	4.6 (5.0)	.14
Peak AEC, cells/μL^f^	8.7 (9.2)	12.2 (13.2)	7.5 (7.3)	.07
Tryptase, ng/mL	7.5 (5.1)	7.2 (5.5)	7.6 (5.0)	.80
Creatinine, mg/dL	1.3 (2.0)	1.9 (2.7)	1.2 (1.7)	.18
% LVEF (%)	61.3 (11.5)	62.0 (12.4)	60.9 (11.2)	.76
Spleen size, cm	10.6 (2.8)	11.6 (3.4)	10.3 (2.5)	.08

Abbreviations: AEC, absolute eosinophil count; BMT, bone marrow transplant; CHIP, clonal hematopoiesis of indeterminate potential; HES, hypereosinophilic syndrome; LT, leukemic transformation; LVEF, left ventricular ejection fraction; NGS, next-generation sequencing; VAF, variant allele frequency.

^a^ *PDGFRA*, *PDGFRB*, and *FGFR1*.

^b^ Clonal hematopoiesis of indeterminate potential–associated genes include *DNMT3A*, *TET2*, *ASXL1*, and *JAK2*.

^c^ Splicing genes include *SF3B1*, *SRSF2*, *U2AF1*, and *ZRSR2*.

^d^ If multiple variations, highest variant allele frequency used.

^e^ To convert to ×10^9^ per liter, multiply by 0.001.

^f^ Peak absolute eosinophil count defined as the highest recorded absolute eosinophil count in our medical system at any time.

Laboratory values, including initial and peak absolute eosinophil count, tryptase, blood counts (data not shown), and hematologic parameters, did not differ between patients with and without thrombotic events. There was no significant difference in specific treatments between patients with and without thrombotic events.

### Association of Molecular Aberrations With Thrombosis and Death in HES

Sixty-four of 71 patients (90%) had NGS testing performed, and 24 patients (34%) had molecular aberrations noted (4 patients [6%] with fusion events, 1 [1%] with 20q chromosomal deletion, and 19 [27%] with at least 1 gene variation on NGS). Significantly more patients with thrombotic events had at least 1 molecular aberration compared with those without thrombotic events (12 of 17 [71%] vs 12 of 54 [26%]; *P* = .003). Four of 71 patients (6%) with molecular aberrations had fusions involving the *PDGFRA*, *PDGFRB*, or *FGFR1* genes, and no thrombotic events occurred in that patient subset. Variations in genes associated with CHIP (5 of 17 patients [29%] vs 3 of 54 patients [6%]; *P* = .03) and with splicing (5 of 17 patients [29%] vs 0, *P* < .001) were significantly more frequent in patients with thrombotic events compared to those without thrombotic events (Table 2).

Thrombotic events occurred in 12 of 24 patients (50%) with molecular aberrations (including patients with gene fusions and variations on NGS) compared with 5 of 40 patients (13%) without (Table 3). Patients with molecular aberrations had significantly higher rates of VTE (9 of 24 [38%] vs 2 of 40 [5%]; *P* = .002) but not arterial events (5 of 24 [21%] vs 3 of 40 [8%]; *P* = .14). Death was more common in patients with molecular aberrations compared with those without (6 of 24 [25%] vs 1 of 40 [3%]; *P* = .009). Among patients with variations associated with CHIP (*DNMT3A, TET2, ASXL1, JAK2*), thrombosis occurred in 5 patients (62%; odds ratio [OR], 5.6; 95% CI, 1.3-15.9) and death in 3 patients (37%; OR, 6.1; 95% CI, 1.3-26.9). Similarly, among patients with spliceosome variations (*SF3B1* [OMIM 605590], *SRSF2* [OMIM 600813], *U2AF1* [OMIM 191317], *ZRSR2* [OMIM 300028]), 5 had a 100% thrombosis event rate (OR, ∞; 95% CI, 4.7-∞), and 4 had an 80% death rate (OR, 55.0; 95% CI, 5.6-674.0). Among patients with molecular aberrations, there was no difference in variant allele frequency between the patients who had thrombosis (mean [SD], 44.2% [25.1%]) compared with those who did not (mean [SD], 26.6% [17.1%]). However, patients with more than 1 variation were more likely to have a thrombotic event compared with patients without thrombosis (7 of 17 [58%] vs 0; *P* = .002). Kaplan-Meier analysis for thrombotic event-free and death-free survival for patients with vs without molecular aberrations is shown in Figure A and B. Comutational analysis is shown in eFigure 2 in the Supplement.

**Table 3.** Outcomes by Molecular Aberration Status

Characteristic	No. (%)			*P* value
	All (N = 71)	Aberration		
		Molecular (n = 24)	No (n = 40)	
Death	8 (11)	6 (25)	1 (3)	.009
Thrombotic events				
Any	17 (24)	12 (50)	5 (13)	.003
VTE	11 (15)	9 (38)	2 (5)	.002
ATE	8 (11)	5 (21)	3 (8)	.14

Abbreviations: ATE, arterial thrombotic events; VTE, venous thromboembolic event.

**Figure.**
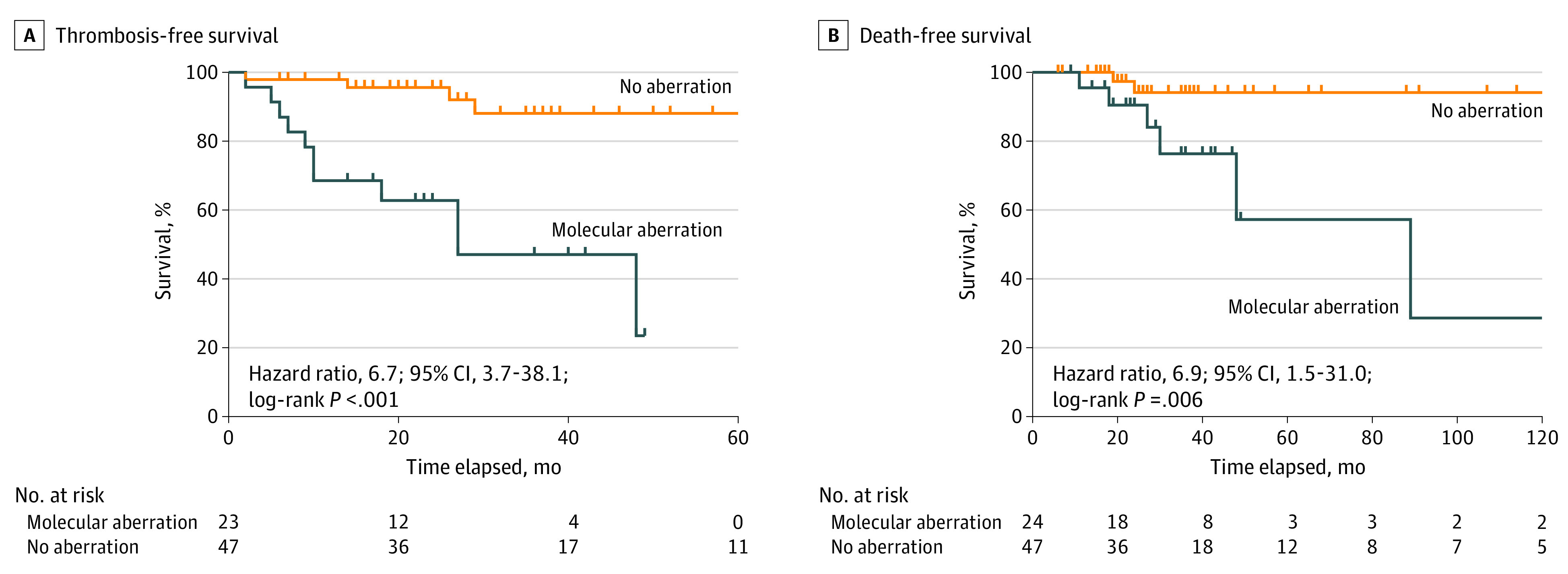
Association of Molecular Aberrations With Thrombosis and Death-Free Survival Kaplan-Meier survival curves of composite outcome of thrombosis-free (including venous and arterial thrombosis) survival (A) and death-free survival (B) in patients with hypereosinophilic syndromes (HESs) who had undergone next-generation sequencing testing (n = 64) with and without molecular aberrations. Dashes represent censored patients (loss to follow-up or death).

### Risk Factors for Thrombosis and Death

Univariate analysis showed that the presence of molecular aberration (OR, 7.0; 95% CI, 2.0-22.0; *P* = .003), ECOG status of 1 or greater (OR, 9.3; 95% CI, 2.7-29.6; *P* < .001), cardiac involvement (OR, 5.6; 95% CI, 1.5-17.6; *P* = .01), and baseline aspirin use (OR, 3.9; 95% CI, 1.2-11.5; *P* = .03) were all associated with increased thrombotic risk. A multivariable logistic regression model using age, CVD, ECOG status, cardiac involvement, hypertension, aspirin use, and presence of molecular aberration as covariates was used to study the simultaneous association of all risk factors with the risk of thrombosis. The presence of molecular aberration (adjusted OR, 5.4; 95% CI, 1.1-27.7; *P* = .04) and ECOG status of 1 or greater (adjusted OR, 12.0; 95% CI, 2.1-69.4; *P* = .005) were associated with significantly increased risk of thrombosis. Age, CVD, cardiac involvement, aspirin use, and hypertension were not associated with increased risk of thrombosis. Univariate analysis also showed that the presence of molecular aberration (OR, 13.0; 95% CI, 1.8-152.2; *P* = .009), history of CVD (OR, 7.5; 95% CI, 1.6-38.0; *P* = .02), ECOG status of 1 or greater (OR, 8.1; 95% CI, 1.7-41.1; *P* = .01), and thrombotic event after HES diagnosis (OR, 14.2; 95% CI, 2.8-72.2; *P* = .002) were associated with an increased risk of death. However, multivariable logistic regression testing utilizing age, CVD, ECOG status, thrombosis, and molecular aberration demonstrated that only thrombotic event after HES (adjusted OR, 74.4; 95% CI, 2.0-2806.0; *P* = .02) and age at HES diagnosis (adjusted OR, 1.2; 95% CI, 1.0-1.5; *P* = .03) were significantly associated with increased odds of death (Table 4).

**Table 4.** Risk Factors for Thrombosis and Death

Variable	OR (95% CI)	*P* value	Adjusted OR (95% CI)	*P* value
Thrombosis^a^				
Molecular aberration	7.0 (2.0-22.0)	.003	5.4 (1.1-27.7)	.04
CVD	2.9 (0.9-8.1)	.08	1.0 (0.2-6.6)	.98
ECOG ≥1	9.3 (2.7-29.6)	<.001	12.0 (2.1-69.4)	.005
Cardiac involvement	5.6 (1.5-17.6)	.01	3.7 (0.5-25.6)	.18
Hypertension	3.1 (1.0-9.1)	.05	4.4 (0.4-44.0)	.21
Baseline aspirin	3.9 (1.2-11.5)	.03	2.8 (0.1-102.3)	.58
Death^b^				
Thrombotic event	14.2 (2.8-72.2)	.002	74.4 (2.0-2806.0)	.02
Molecular aberration	13.0 (1.8-152.2)	.009	0.9 (0.1-12.8)	.97
CVD	7.5 (1.6-38.0)	.02	10.3 (0.6-180.0)	.11
ECOG ≥1	8.1 (1.7-41.1)	.01	4.3 (0.4-52.0)	.25

Abbreviations: CVD, cardiovascular disease; ECOG, Eastern Cooperative Oncology Group; OR, odds ratio.

^a^ Adjusted for age, cardiovascular disease, ECOG score, cardiac involvement, hypertension, aspirin use, and molecular aberrations.

^b^ Adjusted for age, cardiovascular disease, ECOG score, thrombosis, and molecular aberrations.

## Discussion

Hypereosinophilic syndromes are a heterogeneous and rare group of disorders. Treatment is usually aimed at preventing organ damage from eosinophilic infiltration. However, in this cohort study, we found an increased thrombotic risk that has not been previously characterized. Our results suggest that thrombosis prevention was not consistently considered in the treatment algorithm for these patients. We observed a high incidence of VTEs and ATEs in patients with HES, which were associated with a worse prognosis and significantly increased risk of death. We also observed risk factors that may be associated with the risk of thrombosis in this patient population, including the presence of molecular aberrations on NGS, which was associated with an approximately 5-fold increased risk of thrombosis after multivariable analysis. We also observed an association of several risk factors with thrombosis in this patient population, including ECOG status of 1 or greater. Although aspirin use was associated with an increased risk of thrombosis in our univariate analysis, multivariable analysis did not demonstrate an association between aspirin and thrombosis. The increased rates of aspirin use in patients with thrombosis may reflect the increased presence of CVD in that patient group. Interestingly, after multivariable analysis, neither aspirin use nor CVD was associated with increased thrombotic risk. Additionally, having at least 1 thrombotic event after HES diagnosis was found be significantly associated with increased risk of death after multivariable analysis.

The interaction between genetic variations and risk of thrombosis and mortality is an area of active investigation in solid malignant neoplasms and disorders of clonal hematopoiesis including CHIP and myeloproliferative neoplasms.^15,19^ We showed that having at least 1 molecular aberration increases the risk of thrombosis. However, not all variations were associated with the same outcomes in our small study. Notably, our study demonstrated variations commonly associated with CHIP (including *DNMT3A, TET2, ASXL1,* and *JAK2)* and spliceosome genes (*SF3B1, SRSF2, U2AF1, ZRSR2)* were associated with high rates of thrombosis and death. Variations in spliceosome genes in patients with myeloproliferative neoplasms and myelodysplastic syndrome have been known to be associated with higher mortality, increased risks of progression to fibrosis, and leukemia.^20,21^ However, there is a paucity of studies investigating the thrombotic risk of spliceosome gene variations in thrombosis, and it is an area worthy of further exploration. Although the presence of molecular aberrations was also associated with increased risk of death in univariate analysis, our multivariable model showed that the increased risk of death in patients with molecular aberrations may be explained by the increased rates of thrombosis.

### Limitations

Limitations of our study were largely due to our small sample size and the retrospective nature of the study; many patients had undergone HES work-up before the use of molecular testing, particularly NGS. Additionally, it was unclear whether molecular aberrations aside from those known to be associated with clonal expansion of eosinophils (*PDGFR* and *FGFR1* fusions and *JAK2* variations) present in our cohort of patients with HES represented actual clonal expansion vs concomitant CHIP. Notably, our cohort had 8 patients (33% of patients with molecular aberrations) with variations associated with CHIP (*JAK2*, *TET2*, and *DNMT3A*), which is higher than the 10% prevalence noted in previous studies.^15^ Although the mechanisms of increased thrombosis in patients with CHIP-associated variations have been investigated, the mechanisms behind the potential prothrombotic influence of spliceosome genes remain unexplored. Although we did use a multivariate logistic regression model to ascertain risk of thrombosis in patients with molecular aberration, our sample size was small, and we were not able to control for all known and unknown confounders.

## Conclusions

In this cohort study, we observed a high incidence of thrombotic events in patients with HES. We observed an association between identified molecular aberrations on NGS and thrombosis, such that patients with a molecular aberration had an approximately 5-fold increased risk of thrombosis compared with those without. We also observed an association between thrombosis and death, independent of other relevant variables. These results suggest a possible utility of genetic testing for somatic variations in patients with newly identified HES in order to identify patients at high risk of thrombosis and death who might benefit from thromboprophylaxis. Future investigation in larger cohorts is warranted to further investigate the risk of thrombosis in patients with HES and the potential role of thromboprophylaxis in this patient population.

## Notes

Supplement. **eFigure 1.** Patient Flow Chart **eFigure 2.** Comutation Plot of Genes Mutated on NGS Testing and Their Association With Thrombotic Events
