# Supplementary material for: Association of Thrombosis With Hypereosinophilic Syndrome in Patients With Genetic Alterations
Source: JAMA Netw Open. 2021 Aug 6;4(8):e2119812. doi: 10.1001/jamanetworkopen.2021.19812 (PMC8346937; doi:10.1001/jamanetworkopen.2021.19812)
Supplement: Supplement. — eFigure 1. Patient Flow Chart eFigure 2. Comutation Plot of Genes Mutated on NGS Testing and Their Association With Thrombotic Events [file jamanetwopen-e2119812-s001.pdf]

Supplementary Online Content

Leiva O, Baker O, Jenkins A, et al. Association of thrombosis with hypereosinophilic syndrome in patients with genetic alterations. *JAMA Netw Open*. 2021;4(8):e2119812. doi:10.1001/jamanetworkopen.2021.19812

eFigure 1. Patient Flow Chart

eFigure 2. Comutation Plot of Genes Mutated on NGS Testing and Their Association With Thrombotic Events

This supplementary material has been provided by the authors to give readers additional information about their work.

© 2021 Leiva O et al. *JAMA Network Open.*

eFigure 1. Patient Flow Chart

Supplemental Figure 1

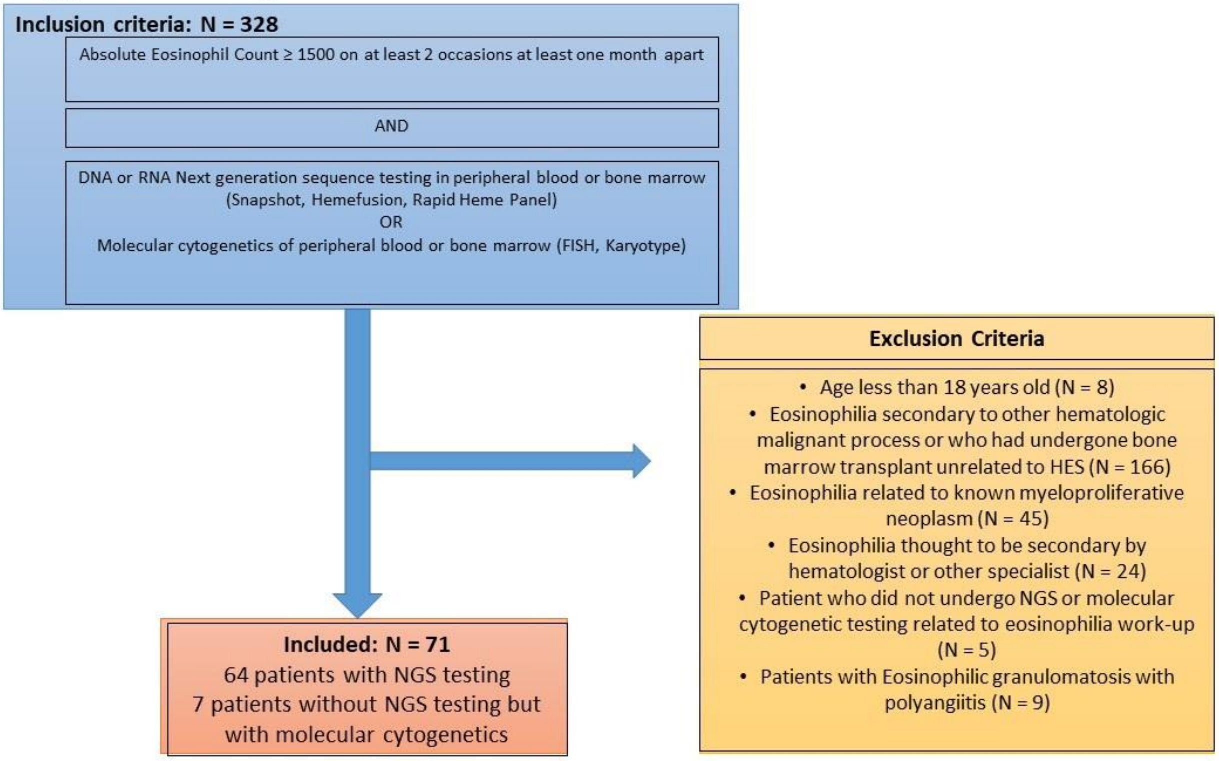

eFigure 2. Comutation Plot of Genes Mutated on NGS Testing and Their Association With Thrombotic Events

Supplemental Figure 2

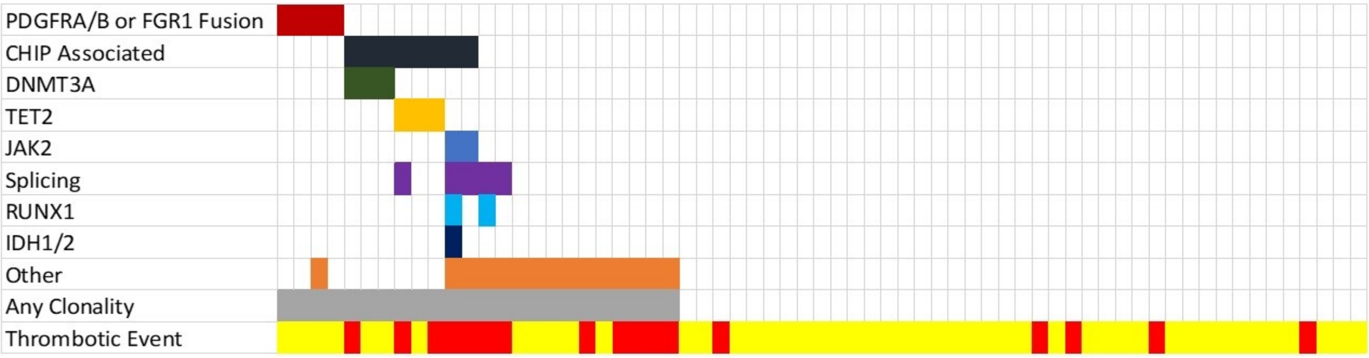

Co-mutation plot of genes mutated on NGS testing and their association with thrombotic events (red rectangles).

© 2021 Leiva O et al. *JAMA Network Open.*
